# Associations between PM_2.5_, ambient heat exposure and congenital hydronephrosis in southeastern China

**DOI:** 10.3389/fpubh.2024.1389969

**Published:** 2024-07-29

**Authors:** ZhiMeng Huang, XiaoHong Zhong, Tong Shen, SongLei Gu, MengNan Chen, WenLi Xu, RuiQi Chen, JinZhun Wu, XiaoQing Yang

**Affiliations:** ^1^Department Pediatrics, School of Medicine, Women and Children's Hospital, Xiamen University, Xiamen, Fujian, China; ^2^Department Prenatal Diagnosis, School of Medicine, Women and Children's Hospital, Xiamen University, Xiamen, Fujian, China

**Keywords:** congenital hydronephrosis, heat exposure, PM_2.5_, China, Xiamen

## Abstract

**Objectives:**

This research aims to analyze how exposure to fine particulate matter (PM_2._5) and ambient heat during pregnancy increases the risk of congenital hydronephrosis (CH) in newborns.

**Methods:**

A case–control study was conducted to investigate the relationship between exposure to PM_2.5_ and ambient heat during pregnancy and the occurrence of CH in newborns. The study, which was conducted from 2015 to 2020, included 409 infants with CH as the case group and 409 infants without any abnormalities as the control group. Using spatial remote sensing technology, the exposure of each pregnant mother to PM_2.5_ concentration was meticulously mapped. Additionally, data on the ambient temperature of exposure for each participant were also collected. A logistics regression model was used to calculate the influence of exposure to PM_2.5_ and ambient heat on the occurrence of CH. Stratified analysis and interaction analysis were used to study the interaction between ambient heat exposure and PM_2.5_ on the occurrence of CH.

**Results:**

At the 6th week of gestation, exposure to PM_2.5_ may increase the risk of CH. For every 10 μg/m^3^ increase in PM_2.5_ exposure, the risk of CH increased by 2% (95%CI = 0.98, 1.05) at a *p-*value of >0.05, indicating that there was no significant relationship between the results. Exposure to intense heat at 6th and 7th weeks of gestation increased the risk of CH. Specifically, for every 1°C increase in heat exposure, the risk of CH in offspring increased by 21% (95%CI = 1.04, 1.41) during the 6th week and 13% during the 7th week (95%CI = 1.02, 1.24). At 5th and 6th weeks of gestation, the relative excess risk due to interaction (RERI) was greater than 0 at the 50th percentile (22.58°C), 75th percentile (27.25°C), and 90th percentile (29.13°C) of daily maximum temperature (Tmax) distribution, indicating that the risk of CH was higher when exposed to both ambient heat and PM_2.5_ at the same time compared to exposure to a single risk factor.

**Conclusion:**

Exposure to higher levels of PM_2.5_ and ambient heat during pregnancy increases the risk of CH in infants. There was a positive interaction between exposure to intense heat and high concentration of PM_2.5_ on the occurrence of CH.

## Introduction

The occurrence of birth defects can be attributed to many factors, such as advanced maternal age, gestational diabetes, or the use of related drugs, which may lead to an increase in fetal malformations (1–3). Congenital hydronephrosis (CH) is a birth defect and a pathological condition of the renal pelvis and is characterized by calyceal dilatation, which is caused by urine stagnation or reflux. CH may result in clinical manifestations such as recurrent urinary tract infections, hematuria, and hypertension (4). In 2010, the American Urological Association defined CH using a color Doppler ultrasound examination, identifying it when the anteroposterior diameter of the renal pelvis was >4 mm in the second trimester or >7 mm in the third trimester (5). With the change in examination technology and pregnancy environment, the incidence of CH is increasing. According to a survey, the incidence of CH in southern China was 4.86/10,000 persons between 2019 and 2020, ranking fifth among all the birth defects (6). From 1995 to 2004, there were 3,648 cases of CH in more than 20 European countries, with an overall prevalence of 11.5/10,000 persons (7).

Related studies have shown that air pollutants such as O_3_, PM_2.5_, and PM_10_ can increase the incidence of respiratory tract infections, asthma, birth defects, and other diseases, ultimately leading to increased mortality rates (8, 9). In the United States, Choi et al. used a multi-pollutant model and found that exposure to both CO and SO_2_ during pregnancy increased the risk of congenital limb defects [OR=1.23 (1.06, 1.42)] (10). A study on the relationship between PM_2.5_ and congenital heart disease in Wuhan, China, showed that, for every 10 μg/m^3^ change in PM_2.5_ concentration, the risk of congenital heart disease increased by 11–17% (11). A case–control study in Taiwan showed that exposure to high concentrations of PM_2.5_ in the first 3 months of pregnancy increased the incidence of hypospadias [OR = 1.40, 95% confidence interval (95%CI) = (1.08, 1.82)] (12).

This research included the data on newborns in southern China from 2015 to 2020, analyzed the general prevalence of CH, the distribution of PM_2.5_ and temperature in southern China, and examined the relationship between exposure to PM_2.5_ and ambient heat and CH. The objective of this research is to ascertain the occurrence and related causes of CH in southern China.

## Methods

### Data sources and quality control

**Figure 1:**
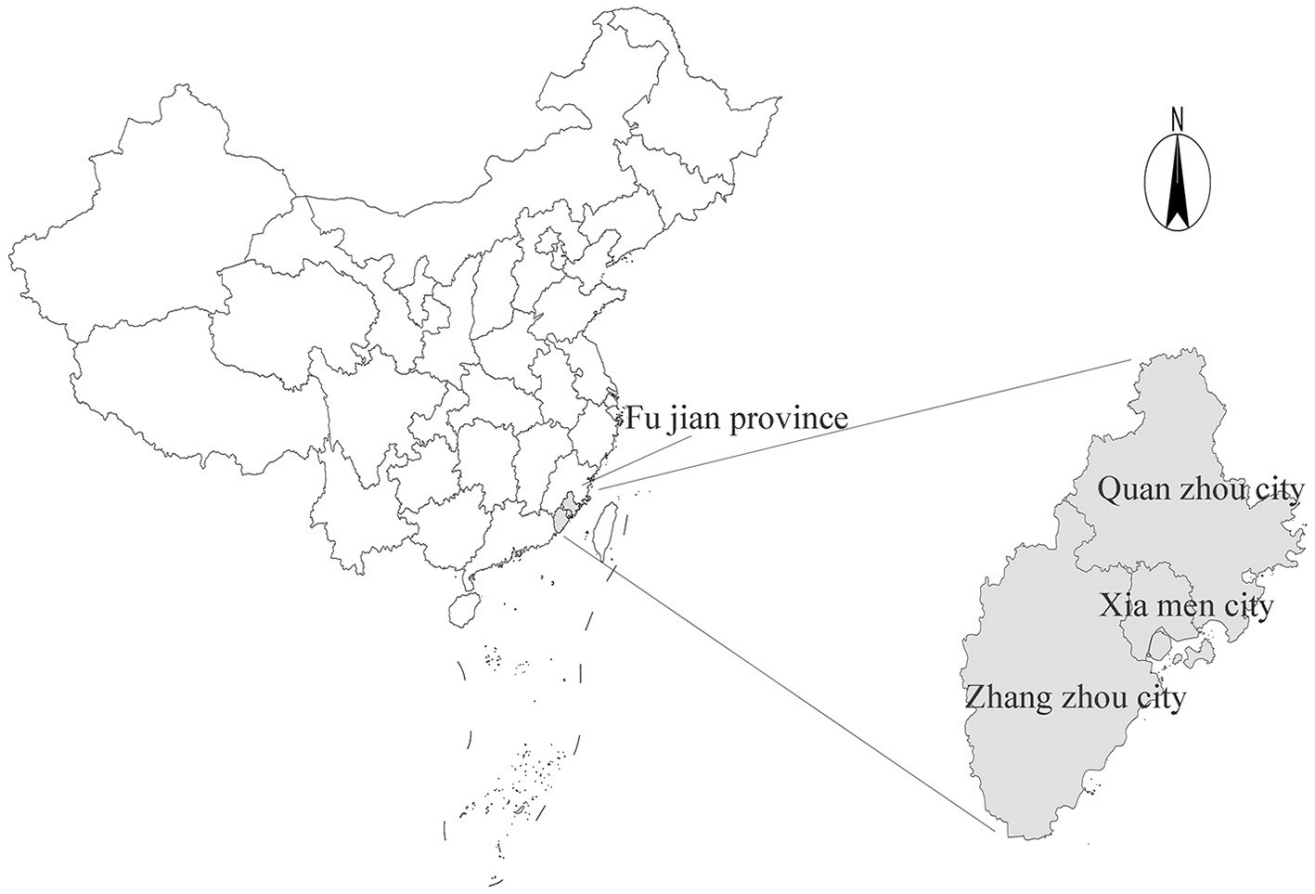
The geographical area studied in this research (shadowed area).

**Figure 2:**
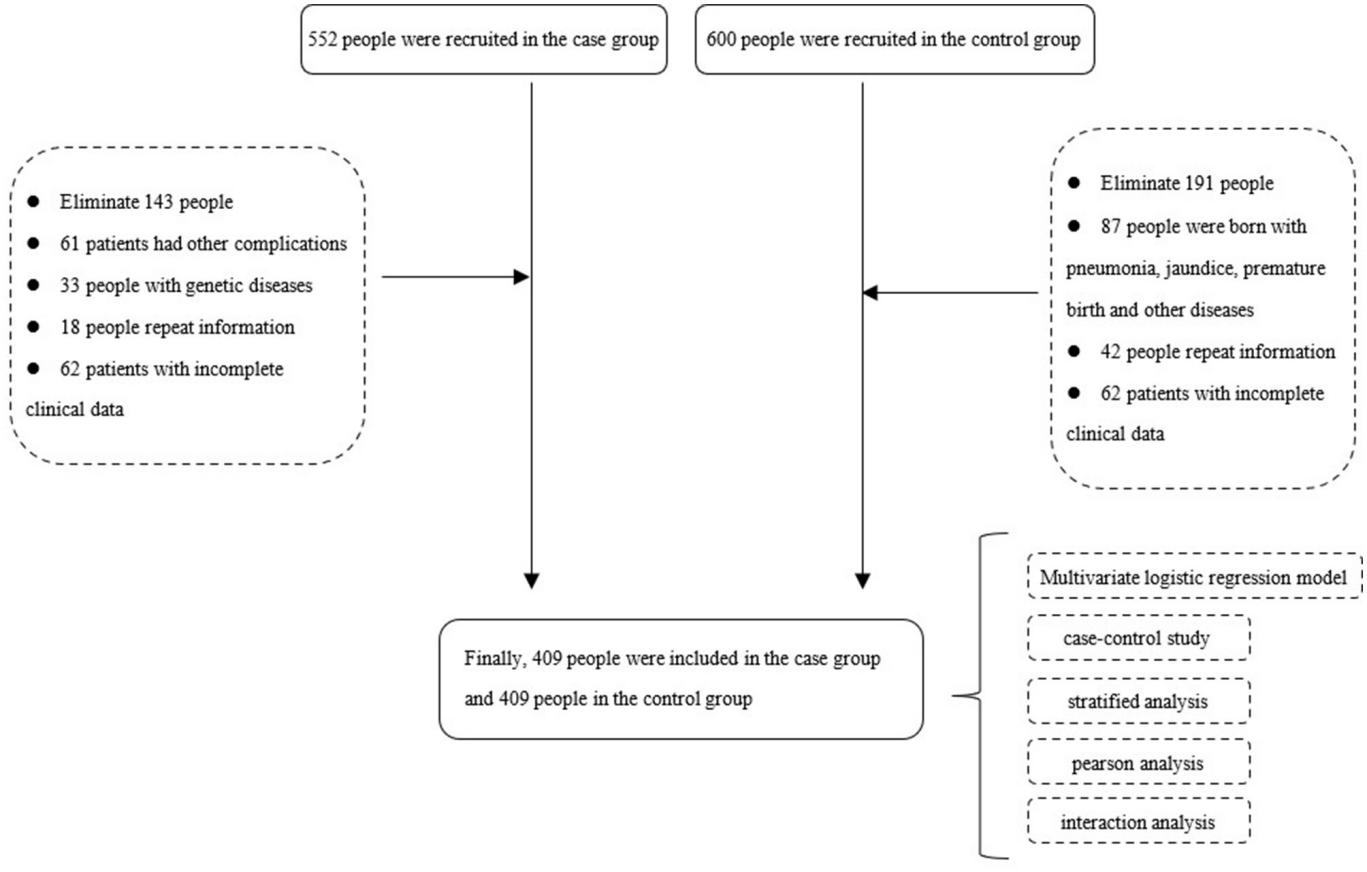
Flowchart of research population recruitment and research methods.

The clinical data of 409 children with CH from 1 January 2015 to 31 December 2020 were obtained from delivery institutions in southern China. CH was diagnosed by obstetricians, pediatricians, or surgeons through ultrasonography, urography, magnetic resonance imaging, and other techniques and clinical manifestations (5). The disease was coded according to the International Classification of Diseases 10 (ICD-10), and the birth defect disease was diagnosed and reported according to the requirements of the “China Birth Defect Monitoring Program” (13). Full-term healthy newborns without any birth defects were used as the control group. Data were collected on several parameters including maternal age, parity, pregnancy disease, past medical history and infant gestational age, fetal sex, and birth time, among others (see Figures 1, 2).

### Exposure assessment

The PM_2.5_ data were obtained from the monitoring data of China's ground air pollution monitoring station and the multi-angle atmospheric corrected aerosol optical depth (AOD) provided by the National Aeronautics and Space Administration of the United States. Combined with meteorological data, land use type, road network information, surface elevation, and pollutant emissions and using satellite remote sensing technology and machine learning methods, a stable PM_2.5_-AOD conversion relationship was constructed using the space–time extremely randomized trees (STETs) model. The daily average PM_2.5_ exposure concentration was determined at a spatial resolution of 1 km. The random 10-fold cross-validation R^2^ value was 0.89 (14, 15).

The original data on ambient temperature were collected from the National Environmental Information Center. By obtaining the daily temperature values of 12,312 meteorological stations, the daily Tmax grid map of the whole country was obtained using the inverse distance weighted interpolation method.

According to the climatic characteristics of the southern region of China, we stipulated that the mothers participating in the study had at least 1 day of exposure to PM_2.5_ and temperature within the susceptibility window of CH during the warm season (May–October) (16, 17). According to the distribution of ambient temperature in 2015–2020, the 10th percentile (14.25°C), 25th percentile (17.54°C), 50th percentile (22.58°C), 75th percentile (27.25°C), and 90th percentile (29.13°C) of Tmax were analyzed using the stratified method (18).

Based on the residential address of the participants during pregnancy, we obtained the geographical coordinates and used ArcMap 10.3 software to perform spatial location matching to calculate the pollutant concentration in the residential area of pregnant women. We thus obtained the PM_2.5_ exposure level and ambient heat exposure at the corresponding spatial location. Relevant literature shows that the critical period of fetal renal pelvis and ureter development is between 5th week and 7th week of gestation. We selected this period for time matching the exposure concentration (19).

### Study design and statistical analysis

**Figure 3:**
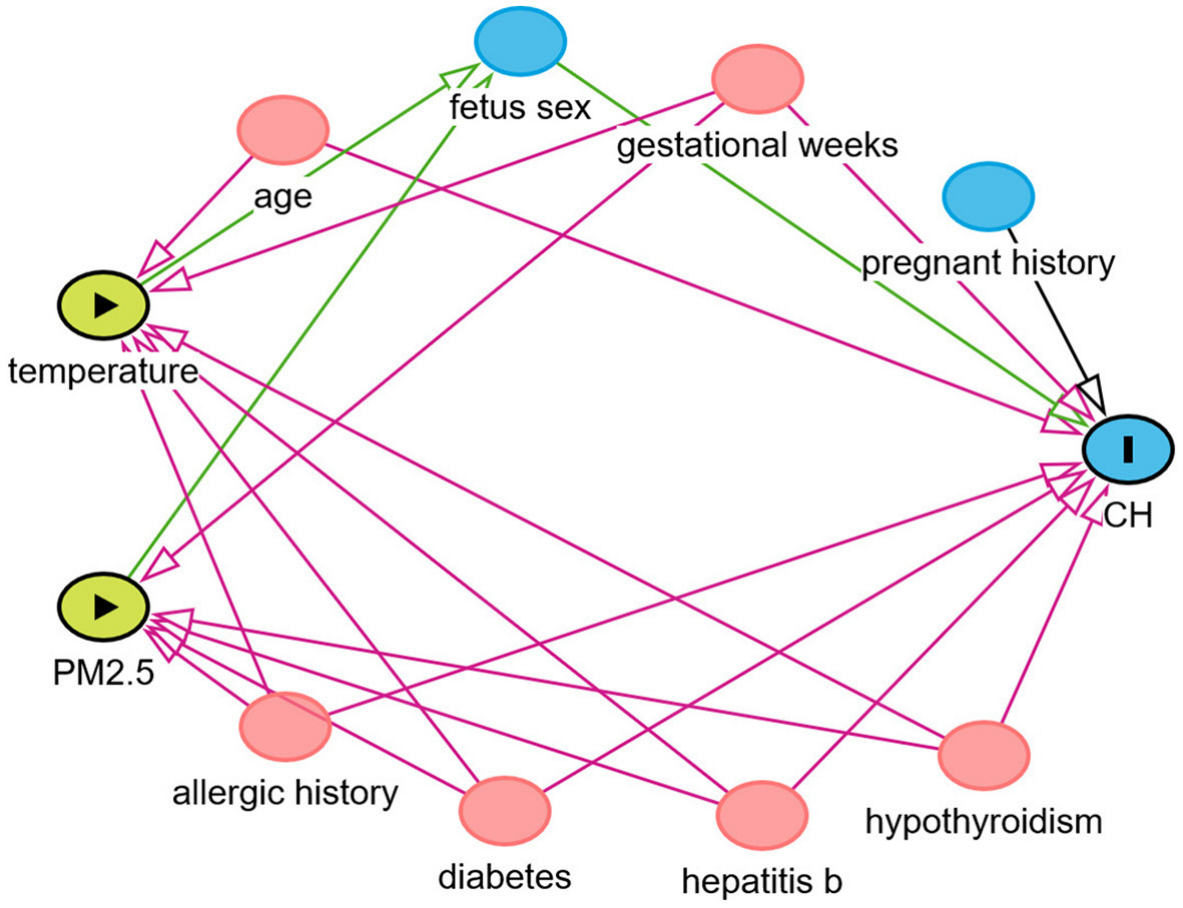
Directed acyclic graph of CH and related risk factors.

Percentile description and the Chi-squared test were performed considering the following confounding factors: maternal age, parity, disease during pregnancy, past medical history, gestational age, and weight of infants. Using the data exposure assessment method, the exact values of PM_2.5_ and temperature were obtained for each participant. A multivariate logistic regression model was established to evaluate the relationship between the risk of CH and PM_2.5_ concentration and ambient temperature. Using directed acyclic graphs, we referred to the relevant literature to evaluate confounding factors (20, 21). The following covariates were incorporated into the final model: maternal age, infant sex, pregnancy disease, etc. These confounding factors were analyzed as covariates (22–24) (see Figure 3).

The adjusted odds ratio (aOR) and its respective 95%CI were calculated to represent the association between the risk of CH and exposure to PM_2.5_ and ambient temperature. In addition, we stratified PM_2.5_ with maternal age, fetal sex, pregnancy disease, and other factors.

Finally, the interaction between PM_2.5_ and ambient temperature on CH was evaluated, and the relationship between their combined effects and independent effects was evaluated. We calculated relative excess risk due to interaction (**RERI**), attributable proportion (**AP**), and synergy index (**S**) to evaluate the additive interaction. When RERI and AP were > 0, S was > 1, and *p*-value was < 0.05, the combined effect of air pollutants and intense heat exposure was greater than the sum of independent effects. When RERI and AP were < 0, S was < 1, and *p*-value was < 0.05, there was an antagonistic interaction, which means that when they exist at the same time, PM_2.5_ and intense heat exposure will reduce the interaction (25–27). The incidence of CH (/10,000) was calculated as the number of CH/the number of perinatal infants × 10,000. A value of *p* of < 0.05 indicated that the difference was statistically significant. Statistical analysis was performed using the software program R, version 4.3.0.

## Results

### General situation of CH

A total of 472,241 newborns from 1 January 2015 to 31 December 2020 were considered for the study. Out of them, there were 409 children with CH, and the average incidence of CH was 8.82/10,000 persons. In the case group, 142 pregnant women (34.72%) were older than 30 years and 267 pregnant women (65.28%) were younger than 30 years. The Chi-squared test value **(X**^**2**^**)** of 20.49 (*P* < 0.05) indicated that the difference between different ages was statistically significant. There were 377 cases (68.21%) of full-term infants with gestational age≥37 weeks. The Chi-squared test value (X^2^) of 12.15 (*P* < 0.05) indicated that there was a statistical significance between different gestational weeks. There were 115 cases (6.51%) of gestational diabetes during pregnancy. The Chi-squared value (X^2^) of 44.77 (*P* < 0.05) indicated that gestational diabetes during pregnancy was statistically significant. There were 40 cases (5.91%) of hepatitis B during pregnancy. The Chi-squared test value (X^2^) of 2.31 (*P* > 0.05) indicated that hepatitis B during pregnancy was not statistically significant (see Table 1).

**Table 1:** General prevalence of congenital hydronephrosis.

		**Case group**	**Control group**	**X^2^**	***P***
Age	< 30	142	206	20.49	< 0.05
	≥30	267	203		
Gestational weeks	< 37	32	10	12.15	< 0.05
	≥37	377	399		
Fetal sex	Male	296	238	18.15	< 0.05
	Female	113	171		
Season	Spring	98	136	10.87	< 0.05
	Summer	87	89		
	Autumn	112	100		
	Winter	112	84		
Pregnant history	0	142	163	5.23	>0.05
	1	152	143		
	2	58	63		
	3	58	40		
Artificial insemination	Yes	58	10	34.72	< 0.05
	No	351	381		
Allergic history	Yes	29	18	2.73	>0.05
	No	380	391		
Operation history	Yes	165	78	44.31	< 0.05
	No	244	331		
Diabetes	Yes	115	40	44.77	< 0.05
	No	294	369		
Hepatitis b	Yes	40	28	2.31	>0.05
	No	369	381		
Hypothyroidism	Yes	58	25	14.6	< 0.05
	No	351	384		
Premature rupture of membrane	Yes	73	25	26.71	< 0.05
	No	336	384		

### The time distribution relationship between PM_2.5_ and temperature

**Figure 4:**
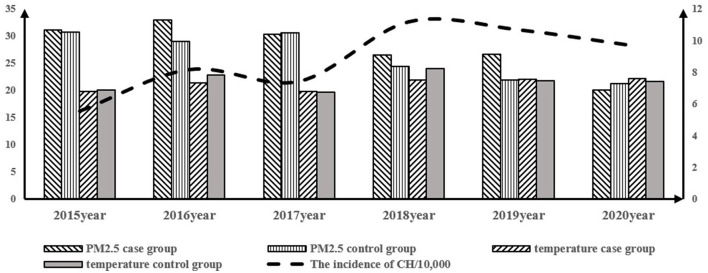
Time distribution of PM_2.5_ and temperature and incidence of CH from 2015 to 2020.

The incidence of CH was 5.57/10,000 in 2015, 8.20/10,000 in 2016, 7.45/10,000 in 2017, 11.21/10,000 in 2018, 10.71/10,000 in 2019, and 9.75/10,000 in 2020. In the case group from 2015 to 2020, the highest temperature was recorded in 2020 (22.18°C) and the highest PM_2.5_ concentration was recorded in 2016 (33.05 μmol/L) (see Figure 4). The average temperature to which the CH group was exposed to was 21.3 ± 5.45°C, and the average temperature to which the healthy control group was exposed to was 22.43 ± 5.01°C. The average concentration of PM_2.5_ to which the case group was exposed to was 28.31 ± 7.97 μmol/L, and the average concentration to which the control group was exposed to was 26.59 ± 7.19 μmol/L (***P*** < 0.05), indicating that the difference was statistically significant (see Table 2).

**Table 2:** Time distribution of PM_2.5_ and temperature.

		**Case group**	**Control group**	***P***
PM_2.5_	Mean ± SD	28.31 ± 7.97	26.59 ± 7.19	< 0.05
	Median	28.15	25.31	
	Range (min-max)	10.76–52.66	12.83–50.69	
	IQR	22.27–33.92	21.90–32.04	
Temperature	Mean ± SD	21.3 ± 5.45	22.43 ± 5.01	< 0.05
	Median	21.69	22.28	
	Range (min-max)	10.94–30.16	11.07–29.46	
	IQR	16.16–26.74	17.64–27.26	

SD, standard deviation; IQR, interquartile range.

### Correlation analysis of PM_2.5_, meteorological factors, and CH

A Pearson correlation analysis was performed on exposure to PM_2.5_, meteorological factors, and CH throughout pregnancy. The results showed that there was a negative correlation between temperature and humidity (*P* < 0.05), and the correlation coefficient was −0.77. There was a negative correlation between precipitation and temperature (*P* < 0.05), and the correlation coefficient was −0.79. We found that precipitation was positively correlated with humidity (*P* < 0.01), and the correlation coefficient was 0.99. There was no correlation between PM_2.5_ and meteorological factors (*P* > 0.05) as well as between CH and meteorological factors (see Table 3).

**Table 3:** Correlation analysis of PM_2.5_, meteorological factors, and CH.

	**PM_2.5_**	**Temperature**	**Humidity**	**Rainfall**	**Wind velocity**	**Number of CH**
PM_2.5_	1	−0.83	0.37	0.37	−0.09	−0.37
Temperature		1	−0.77^*^	−0.79^*^	0.60	0.31
Humidity			1	0.99^**^	−0.83	0.030
Rainfall				1	−0.83	0.028
Wind velocity					1	0.025
Number of CH						1

^*^P < 0.05; ^**^P < 0.01.

### Stratified analysis of CH and pregnancy-related factors under PM_2.5_ exposure

**Figure 5:**
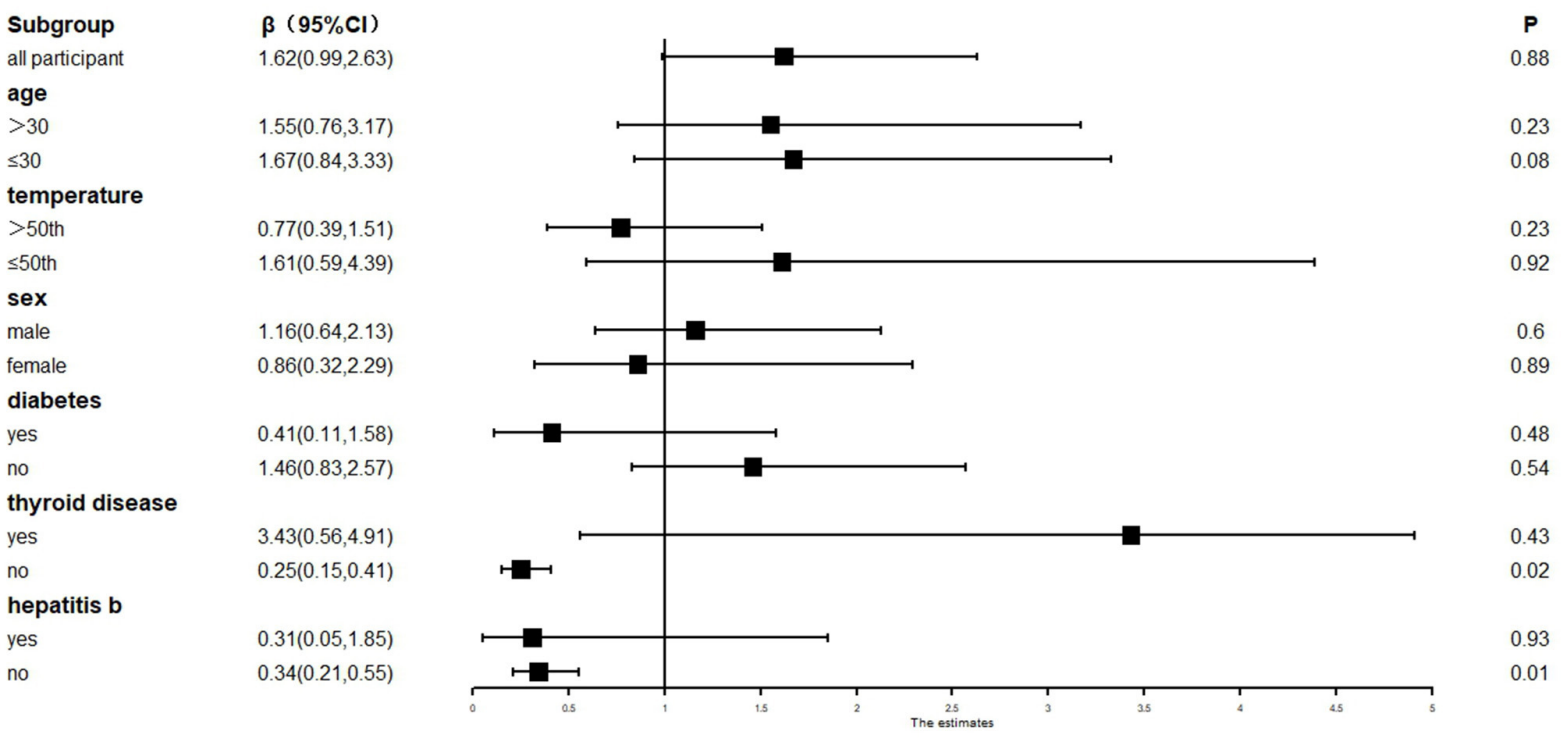
Stratified analysis of CH and pregnancy-related factors under PM_2.5_ exposure. **β**, correlation; 95%CI, 95% confidence interval.

Fetal sex, maternal age, gestational diabetes, and other factors were used for the stratification analysis. The results showed that the presence of thyroid disease and hepatitis B during pregnancy were risk factors for CH in newborns. Pregnant women without thyroid disease and hepatitis B during pregnancy carried a lower risk of CH in newborns [**β** = 0.25 and 0.34, 95%CI = (0.15, 0.41) and (0.21, 0.55)]. No correlation was found between age, fetal sex, gestational diabetes, and CH (see Figure 5).

### The relationship between PM_2.5_, ambient heat exposure, and CH

The critical period of renal pelvis and ureter development is between 5th and 7th weeks of gestation. The correlation between PM_2.5_, temperature, and CH was calculated by using this period of time as the exposure window (19). The results showed that, in the single pollutant model, for every 10 μg/m^3^ increase in PM_2.5_ at the 6th week of gestation, the incidence of CH increased by 1% [OR = 1.01,95%CI = (0.98, 1.10)]. Taking maternal age, fetal sex, pregnancy disease, and other factors as covariates into the analysis, the obtained aOR was 1.02 and the 95%CI was (0.99, 1.05) (*P* > 0.05). In addition, the results revealed that no significant relationship between PM_2.5_ and CH exists.

In the single-pollutant model, we performed a logistic regression analysis of ambient temperature and CH. The results showed that, at 6th and 7th weeks of gestation, for every 1°C increase in temperature, we obtained unadjusted odds ratio (cOR) values of 1.18 [95%CI = (1.05, 1.32)] and 1.12 [95%CI = (1.02, 1.22)], respectively, for CH. The covariate factors such as maternal age, fetal sex, and pregnancy disease were considered in the analysis. The aOR values were 1.18 and 1.13 at the 6th and 7th weeks of gestation, respectively. The 95%CI values at 6th and 7th weeks of gestation were (1.05, 1.33) and (1.03, 1.24), respectively. There was a significant relationship between temperature and CH (see Table 4).

**Table 4:** OR and 95%CI between PM_2.5_, temperature, and CH in 2015–2020.

**Exposure window**	**PM**_**2.5**_		**Temperature**	
	**cOR (95%CI)**	**aOR (95%CI)**	**cOR (95%CI)**	**aOR (95%CI)**
**Single-pollutant model**				
5th week	0.99 (0.96, 1.02)	1.00 (0.95, 1.04)	0.99 (0.90, 1.10)	0.99 (0.91, 1.09)
6th week	1.01 (0.99, 1.05)	1.02 (0.98, 1.05)	1.18 (1.05, 1.32)	1.18 (1.05, 1.33)
7th week	0.97 (0.94, 1.00)	0.97 (0.94, 1.01)	1.12 (1.02, 1.22)	1.13 (1.03, 1.24)
**Two-pollutant model**				
5th week	1.00 (0.96, 1.03)	0.99 (0.96, 1.03)	0.98 (0.89, 1.09)	0.98 (0.88, 1.09)
6th week	1.01 (0.98, 1.06)	1.02 (0.98, 1.05)	1.20 (1.06, 1.35)	1.21 (1.04, 1.41)
7th week	0.97 (0.94, 1.01)	0.97 (0.93, 1.00)	1.14 (1.04, 1.26)	1.13 (1.02, 1.24)

cOR, unadjusted odds ratio; aOR, adjusted odds ratios; 95%CI, 95% confidence interval.

In the two-pollutant model, the environmental temperature and PM_2.5_ were analyzed together with CH. It was found that, at the 6th and 7th weeks of pregnancy, for every 1°C increase in temperature, the cOR was 1.20 and 1.14, respectively. The 95%CI was (1.06, 1.35) at the 6th week of pregnancy and (1.04, 1.26) at the 7th week of pregnancy. Considering the relevant covariates for the analysis, the aOR values were 1.21 and 1.13 at the 6th and 7th weeks of pregnancy, respectively. The 95%CI values were (1.04, 1.41) and (1.02, 1.24) at the 6th and 7th weeks of pregnancy, respectively. There was a significant relationship between temperature and PM_2.5_ on the occurrence of CH (see Table 4).

### Analysis of the interaction between PM_2.5_, ambient heat exposure, and CH

Table 4 shows that the 10th, 25th, 50th, 75th, and 90th of Tmax distribution are used as thresholds for hierarchical analysis. At the 5th week of pregnancy and at the 50th, 75th, and 90th of Tmax distribution, the RERI values were 0.22, 0.46, and 0.60 and the 95%CI values were (0.05, 0.40), (0.01, 0.91), and (0.03, 1.52), respectively, indicating that they had positive additive interaction. The **S** and 95%CI values were 1.04 (1.03, 1.05), 1.07 (1.04, 1.09), and 1.09 (1.04, 1.14) for the 50th, 75th, and 90th of Tmax distribution, respectively, indicating that individuals with both intense heat exposure and PM_2.5_ risk factors had a higher risk of CH than the sum of the risk of single risk factor exposure. At the 6th week of pregnancy, the *p*-value of RERI was < 0.05, indicating that the intense heat exposure and PM_2.5_ had a synergistic effect on the occurrence of CH at the 6th week of pregnancy.

When the Tmax distribution was less than 50th percentile, the 95%CI value of RERI and AP was 0, and the 95%CI of **S** was 1 (*P* > 0.05). PM_2.5_ exhibited no synergistic effect on the occurrence of CH. From these results, it can be indicated that the effect of PM_2.5_ on CH will be enhanced by intense heat exposure (see Table 5).

**Table 5:** The interaction between PM_2.5_, different temperature grades, and CH in 2015–2020.

**Exposure window**	**Temperature grad**	**RERI (95%CI)**	**AP (95%CI)**	**S (95%CI)**	***P***
5th week	< 10th	0.06 (−0.03, 0.14)	0.01 (0.001, 0.02)	1.01 (1.00, 1.02)	>0.05
	25th	0.04 (−0.02, 0.10)	0.01 (0.0003, 0.02)	1.01 (1.00, 1.02)	>0.05
	50th	**0.22 (0.05, 0.40)**	**0.03 (0.02, 0.04)**	**1.04 (1.03, 1.05)**	**< 0.05**
	75th	**0.46 (0.01, 0.91)**	**0.06 (0.03, 0.08)**	**1.07 (1.04, 1.10)**	**< 0.05**
	90th	**0.60 (0.03, 1.52)**	**0.07 (0.03, 0.11)**	**1.09 (1.04, 1.14)**	**< 0.05**
6th week	< 10th	0.03 (−0.05, 0.11)	0.004 (−0.003, 0.01)	1.01 (0.99, 1.01)	>0.05
	25th	0.06 (−0.03, 0.15)	0.01 (−0.0001, 0.02)	1.01 (1.00, 1.02)	>0.05
	50th	**0.42 (0.10, 0.74)**	**0.04 (0.02, 0.05)**	**1.04 (1.03, 1.05)**	**< 0.05**
	75th	**3.25 (0.59, 5.91)**	**0.10 (0.07, 0.12)**	**1.11 (1.09, 1.14)**	**< 0.05**
	90th	**5.46 (0.62, 8.23)**	**0.14 (0.09, 0.18)**	**1.17 (1.11, 1.23)**	**< 0.05**
7th week	< 10th	0.01 (−0.01, 0.03)	0.03 (−0.04, 0.10)	0.98 (0.95, 1.01)	>0.05
	25th	0.01 (−0.01, 0.02)	0.01 (−0.01, 0.03)	0.96 (0.91, 1.01)	>0.05
	50th	0.02 (0.01, 0.04)	0.03 (−0.01, 0.07)	0.95 (0.93, 0.97)	>0.05
	75th	0.03 (0.01, 0.05)	0.04 (−0.01, 0.09)	0.93 (0.91, 0.95)	>0.05
	90th	0.04 (0.01, 0.08)	0.09 (−0.05, 0.22)	0.92 (0.90, 0.95)	>0.05
5-7th weeks	< 10th	0.14 (−0.41, 0.70)	0.02 (−0.04, 0.09)	1.03 (0.96, 1.11)	>0.05
	25th	0.70 (−2.97, 4.37)	0.03 (−0.01, 0.07)	1.03 (0.99, 1.07)	>0.05
	50th	0.32 (−0.73, 1.37)	0.04 (−0.03, 0.11)	1.05 (0.96, 1.13)	>0.05
	75th	1.65 (−3.14, 6.45)	0.09 (0.02, 0.16)	1.10 (1.03, 1.18)	>0.05
	90th	1.66 (−7.29, 10.6)	0.10 (−0.06, 0.26)	1.12 (0.96, 1.32)	>0.05

RERI, relative excess risk due to interaction; AP, attributable proportion; S, synergy index. The bold indicates positive additive interaction.

## Discussion

The early stage of pregnancy, especially 5th−7th weeks of pregnancy, is a critical period for the differentiation and development of the fetal renal pelvis and the ureter (19). We chose this period to study the relationship between PM_2.5_ and ambient temperature and the occurrence of CH. This study shows that exposure to PM_2.5_ and intense heat can lead to increased incidence of CH at ~6th week of gestation, and there is a positive relationship between exposure to intense heat and high concentration of PM_2.5_ and the occurrence of CH.

At present, there are few studies on the etiology of CH. Studies have shown that fetal chromosomal and genetic abnormalities are common causes of malformation in the urinary system (28). Scott et al. performed a clinical follow-up of renal function in 180 children. The results showed that children with congenital thyroid disease had lower renal excretion function than healthy children (29). The results of our stratified analysis showed that mothers without hepatitis B or thyroid dysfunction during pregnancy carried a lower risk of CH in newborns; however, further research is still needed to support this finding.

The results of the global death factors survey in 2017 showed that particulate pollutants caused the global death rate to increase from 4,380,000 to 4,580,000, with China and India among the countries recording the highest number of deaths (30). In 2015, the cost of disease in China due to the population's exposure to high concentrations of PM_2.5_ was a loss of up to RMB ¥1.846 trillion, accounting for 2.73% of China's total annual GDP (31). By comparing the levels of particulate matter pollution on both sides of the placenta under maternal exposure to different concentrations of particulate pollutants, it was found that particulate pollutants could accumulate on the side of the fetus through the placenta (32). In the mouse experimental model, it was found that PM_2.5_ can significantly inhibit the adhesion rate between trophoblast spheres and endometrial epithelial cells by promoting the production of reactive oxygen species (ROS) and can also affect the growth and development of embryos by affecting the expression of long non-coding RNAs (lncRNAs) (33, 34). Padula et al. used the Wald chi-squared test to verify the relationship between 104 genotypes and 5 pollutants. The results showed that high PM_2.5_ could increase the risk of tetralogy of Fallot by mutating the ***SLCO1B1*** fragment gene (35).

Toxicological evidence shows that exposure to higher ambient temperature levels can aggravate the effects of environmental chemicals and increase the possibility of fetal diseases during pregnancy (36). On exposure to extremely high temperature levels, pregnant women are prone to dizziness, fainting, migraine, and aggravation of the original disease. These conditions cause changes in hormone levels in the body, thereby increasing the nutritional needs of the fetus and the mother, ultimately leading to adverse pregnancy outcomes (37, 38). Research has analyzed the relationship between intense heat exposure and birth defects. The results showed that intense heat exposure may lead to an increase in the incidence of congenital heart defects, neural tube defects, oral and facial cracks, renal dysplasia, and other diseases (39).

We analyzed the relationship between the average temperature and PM_2.5_ concentration in different years and the incidence of CH, as shown in Figure 4. It was observed that the incidence of CH gradually increased over time and reached a peak in 2018. Improvement in the detection technology and the completeness of the follow-up system could have led to the detection of CH, but the correlation between PM_2.5_ and CH cannot be verified. Therefore, we accurately assessed the exposure of temperature and PM_2.5_ for each participant and subsequently performed a logistics regression analysis, which helped analyze the relationship between pollutant exposure concentration and CH (Table 4). The results showed that exposure to ambient heat increased the occurrence of CH at the 6th and 7th weeks of gestation.

The Pearson correlation analysis showed that there was a negative correlation between temperature and PM_2.5_, but the difference was not statistically significant. While relevant studies on the relationship between ambient temperature and PM_2.5_ and congenital heart disease are available, there is no research report on the impact of PM_2.5_ and temperature on the incidence of CH. A multi-center study conducted in the United States showed that heat exposure could enhance the effect of PM_2.5_ on the occurrence of ventricular septal defect. The results showed that exposure to the same concentration of PM_2.5_ during pregnancy leads to a higher risk of ventricular septal defect due to intense heat exposure, compared to exposure to a low-temperature environment [OR = 2.14, 95%CI = (1.19, 3.38)] (40). This finding is consistent with the results of our study. Exposure to intense heat will increase the risk of CH under the same concentration of PM_2.5_.

Similarly, Wen Jiang et al. used the recent monitoring station method and the city's average method to study the effects of exposure to air pollutants and intense heat exposure on congenital heart disease in early pregnancy. They found that exposure to CO, NO_2_, SO_2_, PM_2.5_, and O_3_ in early pregnancy increases the risk of congenital heart disease, and environmental thermal exposure exacerbates the impact of these air pollutants (18). Another study conducted in Guangdong, China, showed that the risk of congenital heart disease increased when mothers were exposed to a smoking environment and other environmental pollutants during pregnancy, and there was a significant dose–response relationship. We also found that the risk of CH increased when pregnant mothers were exposed to high temperature and PM_2.5_ at the same time (41).

### Importance of the study and its limitations

This study has some strengths. In this study, CH was professionally managed. The trained nurses were asked about the prenatal exposure factors and the professional doctors used the relevant scales to carry out the preliminary classification of the disease and the reclassification of the severity (42, 43). Inspection and analysis were carried out by relevant statisticians. This study had a large sample size and covered a wide area, encompassing a large population in southern China, which is conducive to the assessment of risk factors. The large sample size also improves the reliability and representativeness of this study. The personal history, disease history, and past history of the mother and the infant were used as covariates to adjust and reduce the interference of other factors. We used spatial remote sensing technology combined with machine learning methods to accurately map PM_2.5_ exposure during pregnancy to the residential address of each pregnant woman, thereby generating accurate air pollutant exposure values. Finally, this study included the medical history data of live births and stillbirths.

Our current research also has some limitations. First of all, our research is based on the living address of pregnant women, but pregnant women spend most of their time at home, and there may be movement during pregnancy, which will affect the accuracy of the results (44). Second, in addition to meteorological factors and air pollution, the influencing factors of CH also include living environment, diet, exercise, and other factors during pregnancy (45–47). However, these factors were not collected for correlation analysis. In terms of statistics, our study was limited by data, and the onset time was limited to the critical period of renal pelvis and ureter development, which limited the application of research duration and related statistical models. Finally, since this study was retrospective in nature, there is a possibility of some recall bias.

## Conclusion

Through the professional management of CH, a detailed analysis of prenatal exposure factors, such as PM_2.5_ and ambient temperature, was performed in this study. Using advanced spatial remote sensing technology, accurate air pollutant exposure values were mapped to each participating mother's address. In the 6th week of pregnancy, exposure to PM_2.5_ and intense heat increases the risk of CH. At the 5th and 6th weeks of pregnancy, simultaneous exposure to intense heat and a high concentration of PM_2.5_ had a positive interaction effect on the occurrence of CH.

## Data availability statement

The raw data supporting the conclusions of this article will be made available by the authors, without undue reservation.

## Ethics statement

The studies involving humans were approved by the Medical Ethics Committee of Xiamen University Women's and Children's Hospital. The studies were conducted in accordance with the local legislation and institutional requirements. The participants provided their written informed consent to participate in this study. The manuscript presents research on animals that do not require ethical approval for their study.

## Author contributions

ZH: Conceptualization, Data curation, Writing – original draft, Writing – review & editing. XZ: Data curation, Methodology, Writing – review & editing. TS: Conceptualization, Investigation, Methodology, Writing – review & editing. SG: Conceptualization, Data curation, Methodology, Supervision, Writing – review & editing. MC: Conceptualization, Investigation, Writing – review & editing. WX: Methodology, Resources, Writing – review & editing. RC: Formal analysis, Writing – review & editing. JW: Methodology, Writing – original draft, Writing – review & editing. XY: Methodology, Resources, Writing – original draft, Writing – review & editing.
